# Fabrication and Characterization of ZnO Nano-Clips by the Polyol-Mediated Process

**DOI:** 10.1186/s11671-018-2458-9

**Published:** 2018-02-09

**Authors:** Mei Wang, Ai-Dong Li, Ji-Zhou Kong, You-Pin Gong, Chao Zhao, Yue-Feng Tang, Di Wu

**Affiliations:** National Laboratory of Solid State Microstructures, Department of Materials Science and Engineering, College of Engineering and Applied Sciences, Collaborative Innovation Center of Advanced Microstructures, Nanjing University, Nanjing, 210093 People’s Republic of China

**Keywords:** ZnO, Nano-clips, Polyol process, Morphology, Growth mechanism

## Abstract

ZnO nano-clips with better monodispersion were prepared successfully using zinc acetate hydrate (Zn(OAc)_2_·nH_2_O) as Zn source and ethylene glycol (EG) as solvent by a simple solution-based route-polyol process. The effect of solution concentration on the formation of ZnO nano-clips has been investigated deeply. We first prove that the 0.01 M Zn(OAc)_2_·nH_2_O can react with EG without added water or alkaline, producing ZnO nano-clips with polycrystalline wurtzite structure at 170 °C. As-synthesized ZnO nano-clips contain a lot of aggregated nanocrystals (~ 5 to 15 nm) with high specific surface area of 88 m^2^/g. The shapes of ZnO nano-clips basically keep constant with improved crystallinity after annealing at 400–600 °C. The lower solution concentration and slight amount of H_2_O play a decisive role in ZnO nano-clip formation. When the solution concentration is ≤ 0.0125 M, the complexing and polymerization reactions between Zn(OAc)_2_·nH_2_O and EG predominate, mainly elaborating ZnO nano-clips. When the solution concentration is ≥ 0.015 M, the alcoholysis and polycondensation reactions of Zn(OAc)_2_·nH_2_O and EG become dominant, leading to ZnO particle formation with spherical and elliptical shapes. The possible growth mechanism based on a competition between complexing and alcoholysis of Zn(OAc)_2_·nH_2_O and EG has been proposed.

**Electronic supplementary material:**

The online version of this article (10.1186/s11671-018-2458-9) contains supplementary material, which is available to authorized users.

## Background

Zinc oxide (ZnO) with a direct wide band gap of 3.37 eV and a large excitation binding energy of 60 meV has attracted great attention in recent years, owing to its applications in photocatalysts, solar cells, and electrical and optical devices [1–10]. ZnO has extremely abundant nanostructures, such as nanospheres, nanorods, nanowires, and nanoflowers [11–16]. Various synthesis methods have been utilized to produce ZnO nanostructures [17–22]. Among these methods, solution-based polyol process exhibits splendid advantages in preparing inorganic compounds (metal, oxide, hydroxyacetate) due to the unique solvents’ characteristics, such as high boiling point (up to 250 °C) and complexing, reducing, and surfactant properties, in addition to their amphiprotic character [23–25]. In the past decades, ZnO nanoparticles with various sizes and morphologies derived from the polyol-mediated approach have been studied extensively. The processing parameters of polyol, reaction temperature and concentration, anion, hydrolysis or alkaline ratio, and additive have great influence on the size and morphology of ZnO particles [11–31]. The spherical oxide particles with the size of 20–500 nm are frequent morphologies when using ethylene glycol (EG) as solvent and Zn(OAc)_2_·2H_2_O as Zn source [23, 28, 30]. The aggregation behavior of the ZnO nanocrystal units to form polycrystalline spheres has been confirmed [18, 24, 26, 27].

In this work, we successfully prepared ZnO nano-clips for the first time by the simple polyol process with zinc acetate hydrate (Zn(OAc)_2_·nH_2_O, *n* < 2) and EG without additional H_2_O or other additives. The effect of solution concentration on morphology has been investigated deeply, and the possible growth mechanism has been proposed.

## Methods

All reagents were of analytical grade and used without further purification. 9.2 mg zinc acetate hydrate (Zn(OAc)_2_·nH_2_O, *n* < 2) was dissolved in 5 mL ethylene glycol (EG) to obtain about 0.01 mol/L (M) colorless solution. The solution was then heated on a hot plate to 170 °C under magnetic stirring for 1–3 h. The solution began to become turbid after 6~7 min with milky floccule formation. While the reaction was over, the precipitate was centrifuged, washed several times at 2000–3000 rpm with ethanol and deionized water (volume ratio of 4:1), and dried in room temperature overnight for structural and morphological characterization. Some samples were also annealed at 400 and 600 °C for 2 h in a tube furnace with a ramp rate of 2 °C/min in air. The solutions with various Zn(OAc)_2_·nH_2_O concentrations of 0.005, 0.125, 0.015, 0.05, and 0.2 M were also prepared so as to investigate the effect of solution concentration.

The crystallinity and phases of the samples were evaluated by an X-ray diffractometer (D/max 2000, Rigaku) with Cu kα radiation (*λ* = 1.5405 Å). The morphological observations were performed by scanning electron microscopy (SEM; Quanta™ 50, FEI) and transmission electron microscopy (TEM; Tecnai G2 F20, Philips). The thermal stability of as-prepared samples was characterized by thermogravimetry-differential thermal gravity analyses (TG-DTG; STA 409 PC, Netszch) in the air flow with a heating rate of 20 °C/min. The Fourier-transform infrared spectra (FTIR) of as-prepared and annealed samples were collected in the 4000–400 cm^− 1^ range with a FTIR spectrometer (FTIR; Spectrum, PerkinElmer) using pressed KBr pellets. The Brunauer-Emmett-Teller (BET) specific surface area was estimated by the surface area apparatus (TriStar-3000, Micromeritics). In addition, the optical property of the annealed sample was also measured via an ultraviolet-visible-near infrared ray (UV–visible–NIR) spectrophotometer (UV-3600, Shimadzu).

## Results and Discussion

### Morphology of ZnO Nano-Clips

**Fig. 1:**
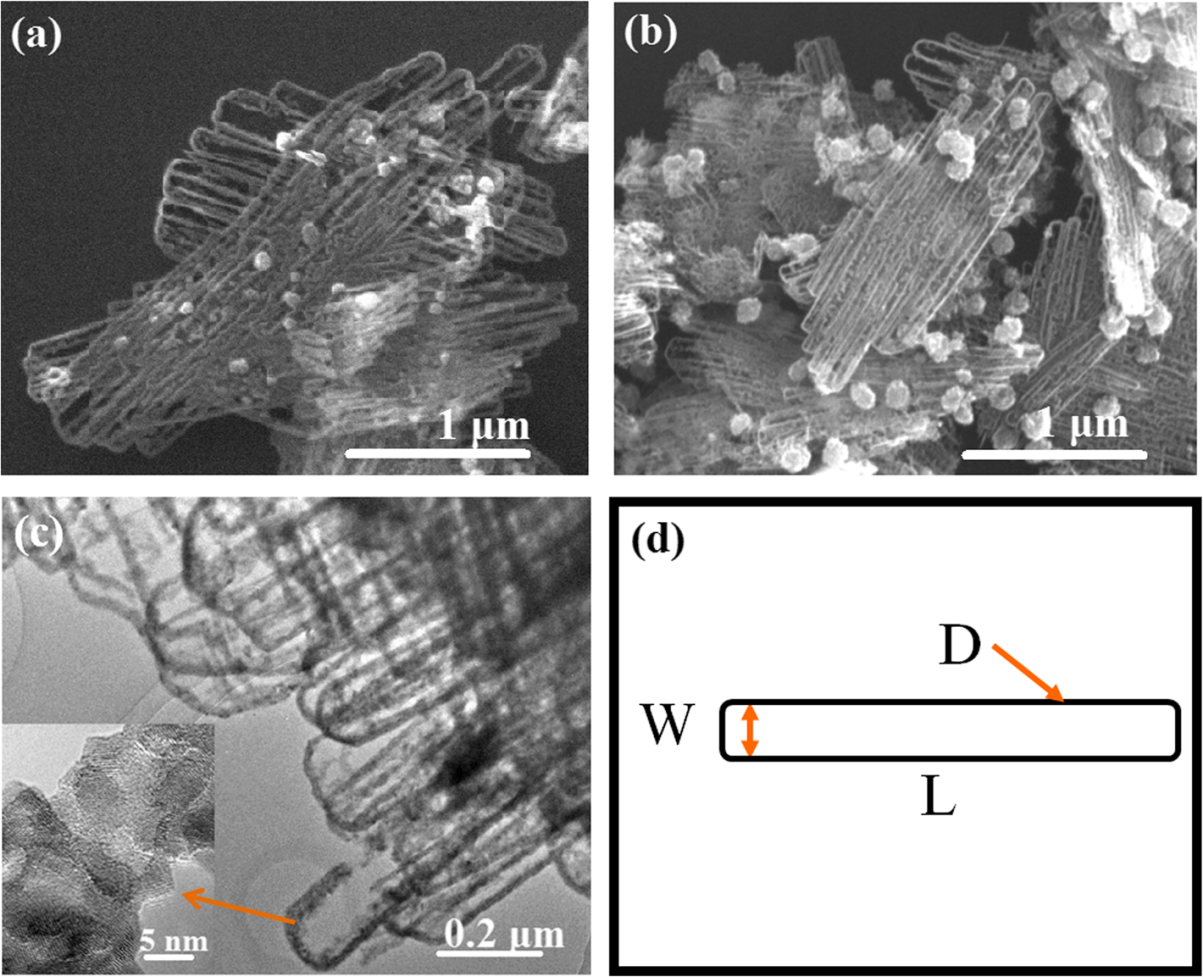
SEM images of (**a**) as-prepared and (**b**) 600 °C annealed ZnO nano-clip samples. TEM images of (**c**) 400 °C annealed ZnO nano-clips. The insets of (**c**) are corresponding HRTEM images of local magnification. (**d**) Sketch diagram of the ZnO nano-clip

Based on some literatures [23, 28, 30], ZnO nanoparticles with spherical or elliptical shapes can be formed in EG solvent using Zn(OAc)_2_·2H_2_O as Zn source at 160 or 198 °C. However, under our processing conditions of 5 mL 0.01 M Zn(OAc)_2_·nH_2_O solution at reaction temperature of 170 °C, 2 h without adding additional H_2_O, ZnO nano-clips with better monodispersion have been fabricated by simple polyol process, as shown in Fig. 1. As-prepared samples exhibit clear clip-like morphology with a large quantity of clips and slight nanoparticles (Fig. 1a). After 600 °C annealing, the morphology basically keeps unchanged (Fig. 1b). We also performed TEM and high-resolution TEM (HRTEM) observations on 400 °C annealed ZnO samples, as seen in Fig. 1c. And the nano-clip morphology can be observed again. Based on the HRTEM pictures of local magnification of 400 °C samples, it can be observed that ZnO clips consist of a lot of aggregated nanocrystals (~ 3 to 15 nm) with polycrystalline structures. Figure 1d depicts the sketch drawing of one ZnO nano-clip with width (W) of 50–100 nm, length (L) of ~ 1–3 μm, and diameter (D) of 10–30 nm. Although ZnO has extremely abundant nanostructures, such morphology like the nano-clip is still very unique and novel, to our knowledge, which has not been reported, especially by a simple polyol-mediated approach.

### Structure of ZnO Nano-Clips

**Fig. 2:**
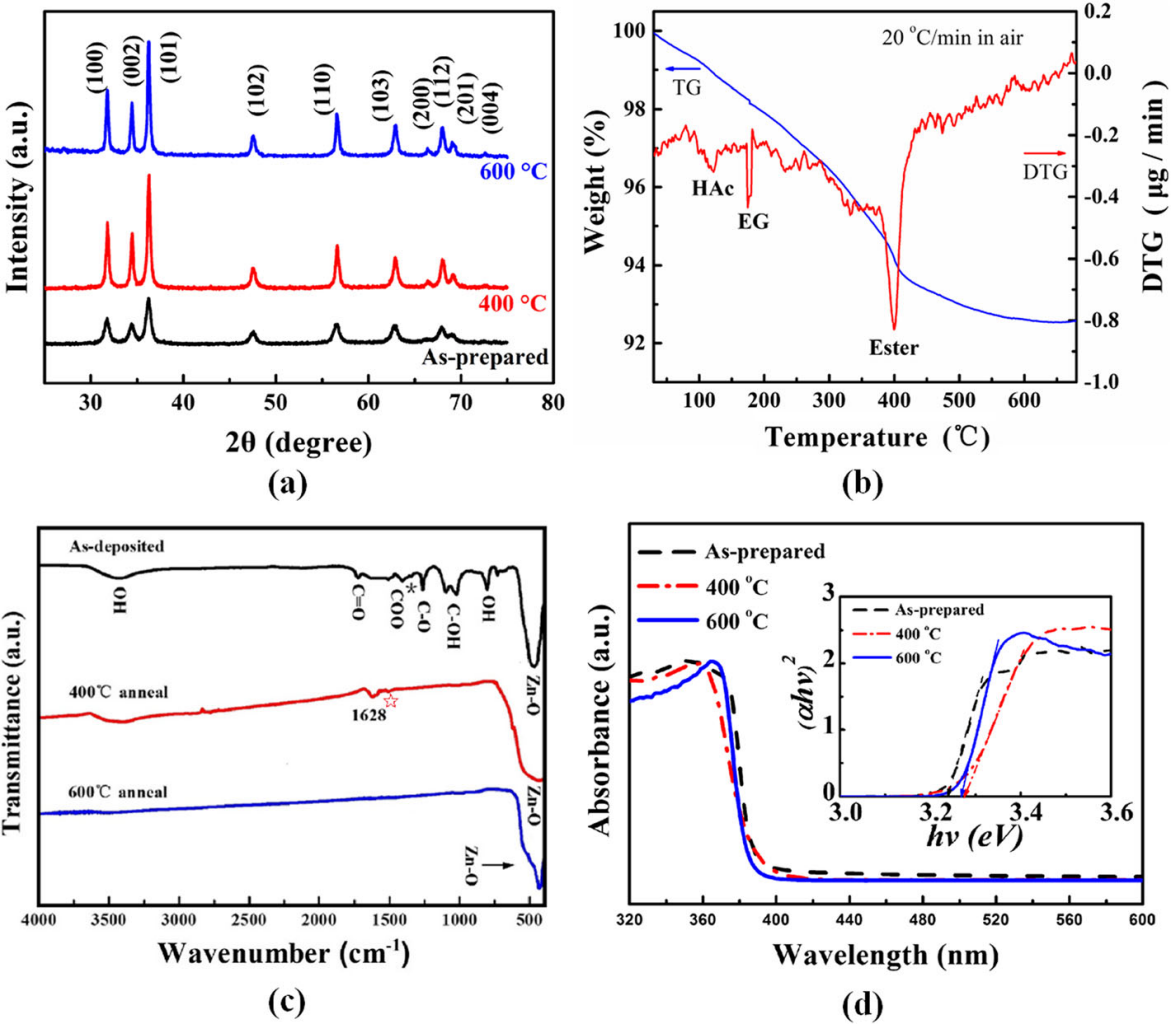
(**a**) XRD patterns of as-prepared, 400 and 600 °C annealed ZnO nano-clip samples. (**b**) TG-DTG curves of as-prepared ZnO nano-clips heated in air. (**c**) FTIR spectra of as-prepared, 400 and 600 °C annealed ZnO nano-clip samples. (**d**) UV–visible absorbance spectrum of as-prepared, 400 and 600 °C annealed ZnO nano-clip samples. The inset in (**d**) is a corresponding curve of (*αhv*)^2^ dependence on *hv*

Figure 2a shows the X-ray diffraction (XRD) patterns of as-prepared, 400 and 600 °C annealed ZnO nano-clips. As-prepared ZnO clips have been mostly crystallized with a hexagonal wurtzite phase (JCPDS36-1451). Quite a few XRD peaks originate from (100), (002), (101), (102), (110), (103), (112), and (201) planes, indicating the polycrystalline nature of ZnO nano-clips, in good agreement with the above HRTEM results (Fig. 1c). After 400 and 600 °C annealing, these XRD ones become stronger and sharper, attesting enhanced crystallinity. Based on the full width at half maximum (FWHM) of three stronger peaks of (101), (100), and (002), the average crystallite size of as-synthesized, 400 and 600 °C nano-clips is calculated to be about 11.5, 21.0, and 24.8 nm, respectively, using the Scherrer equation. Evidently, the annealing significantly improves the crystallinity of ZnO nano-clips and increases the average size of nanocrystals that form nano-clips. However, based on large amounts of SEM observations, there is no significant change in the morphology and size of nano-clips.

Figure 2b records the TG-DTG curves of as-prepared ZnO nano-clips with a heating rate of 20 °C/min in air up to 700 °C. The DTG curve shows three weight loss peaks at around 118, 180, and 400 °C, related to the volatilization of acetic acid and EG, and the severe decomposition and burning of the ester, respectively. The TG curve confirms a small amount (~ 7%) of weight loss from room temperature to 600 °C. After 600 °C, the weight basically is kept unchanged due to the complete removal of organic species in ZnO nano-clips, in accordance with the following FTIR result of ZnO sample annealed at 600 °C (Fig. 2c).

Figure 2c illustrates the FTIR spectra of as-prepared, 400 and 600 °C annealed ZnO nano-clip samples. The as-prepared product shows several absorption bands, which are ascribed to some organic groups or ZnO. The strong adsorption band at 400–600 cm^− 1^ originates from the stretching vibration mode of Zn–O in the low wavenumber region, demonstrating the formation of ZnO. The peak at around 800 cm^− 1^ is assigned to the stretching vibration mode of OH bond in alcohol, and the absorption band in the range of 1020–1090 cm^− 1^ belongs to C–OH bond, which indicates that the as-prepared samples contain a slight amount of polyol. The peaks at 1260 and 1727 cm^− 1^ result from the stretching vibration of C–O and C=O bonds, which implies the presentence of ester or glycolate in as-prepared ones. Two absorption bands at approximately 1587 and 1413 cm^− 1^ correspond to the asymmetric and symmetric stretching vibrations of C=O and C–O in the acetate (COO) groups, respectively [3, 20, 26]. A splitting between the asymmetric and symmetric carboxylate stretching bands (Δ) in the range 130–200 cm^− 1^ is typical of bridging complexes [32]. Herein, the Δ value of 174 cm^− 1^ suggests the bridging bonding mode in as-synthesized ZnO nano-clips. Additionally, the small absorption peak (denoted by *) at 1343 cm^− 1^ is due to weakly bound acetic acid molecules, suggesting that slight acetic acid is adsorbed onto the surface of the as-synthesized ZnO nano-clips, in consistent with the previous reports [11, 26].

After 400 °C annealing, except the extremely weak absorption peak (denoted by ☆) at 1587 cm^− 1^ of C=O, the other IR absorption bands from HAc, ester, and EG have disappeared, in agreement with the TG-DTG results in Fig. 2b. Furthermore, the absorption band at 1628 cm^− 1^ is ascribed to the bending vibration of hydration or water adsorption [26]. The weak broad band in the high wavenumber range of 3440 cm^− 1^ confirms the existence of hydroxyl group in the surface of metal oxide both before and after 400 °C annealing. After 600 °C annealing, the organic compounds and hydroxyl group are removed completely. Only the strong band at 434 cm^− 1^ from Zn–O stretching vibrations can be observed, indicating the pure ZnO formation at 600 °C. The Zn–O peak shift and broadening after 400 and 600 °C annealing might be related to the improved crystallinity, crystallite size, and reduced organic species/impurity.

### Optical Property and Specific Surface Area of ZnO Nano-Clips

Figure 2d shows the UV–visible absorbance spectra of as-prepared, 400 and 600 °C annealed ZnO nano-clip samples. The inset in (d) is the corresponding curves of (*αhv*)^2^ dependence on *hv*. The strong absorption occurs below around 390 nm.

The direct band gap (Eg) of ZnO can be estimated by (*αhv*)^2^ = *c*(*hv* − *Eg*) [33], where *α* is the absorption coefficient and *hv* is the emission photon energy. The calculated bandgap of as-prepared, 400 and 600 °C ZnO samples is 3.24, 3.28, and 3.27 eV, respectively, in consistent with 3.2 eV of ZnO nanoparticles by polyol synthesis [28]. Why does the bandgap increase initially and then slightly decrease with the annealing temperature? We think that several factors will be responsible for this. On the one hand, the bandgap of nanomaterials decreases with increasing the nanocrystal size. On the other hand, the crystalline powders have larger bandgap than the amorphous ones. Meanwhile, the reduced carbon impurity in metal oxide might enhance the bandgap. Based on the XRD and FTIR results, 400 °C ZnO samples have exhibited better crystallinity and lower carbon content. Although the nanocrystal size in 400 °C ZnO nano-clips becomes larger, the evidently improved crystallinity and reduced carbon impurity predominate, which lead to the increased bandgap. When further annealed in 600 °C, the slightly reduced bandgap is mainly ascribed to the grain size effect.

The specific surface area of as-prepared ZnO nano-clip is about 88 m^2^/g. After 400 °C annealing, it decreases to ~ 59 m^2^/g, which is related to the increased crystallite size, the enhanced grain density, and the decreased pores and defects after thermal treatment [26].

### Effect of Solution Concentration on ZnO Morphology

**Fig. 3:**
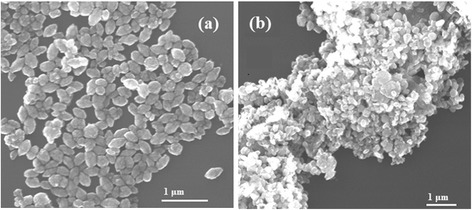
SEM images of ZnO samples under various conditions of (**a**) 0.015 M, 5 mL, and 170 °C and (**b**) 0.05 M, 5 mL, and 170 °C

To investigate the effect of reactant concentration on the formation and morphology of ZnO samples by polyol process, the Zn(OAc)_2_·nH_2_O solution concentration varied from 0.005 to 0.01, 0.0125, 0.015, 0.05, and 0.2 M by fixing other reaction parameters. When the Zn(OAc)_2_·nH_2_O solution concentration is 0.005, 0.01, and 0.0125 M, the ZnO nano-clips can be elaborated with slight nanoparticles, as shown in Fig. 1b. Increasing the solution concentration to 0.015 M, ZnO nano-clips disappear and only ZnO nanoparticles with elliptical shapes (~ 435 × 200 nm) can be formed in Fig. 3a, similar to previous literature results [25, 28, 30]. With further increasing of the solution concentration to 0.05 M, the SEM image shows mixture of elliptical (~ 220–260 × 100–140 nm) or spherical (100–260 nm) particles with several micrometer irregular aggregates in Fig. 3b. Moreover, the reaction becomes rapid with the increment of solution concentration. The solution turbid time shortens from 7 min of 0.01 M to 4.5 min of 0.2 M. The ZnO products of 0.2 M exhibit more messy aggregate morphology with ~ 30-nm small spheres.

### The Possible Growth Mechanism of ZnO Nano-Clips

**Fig. 4:**
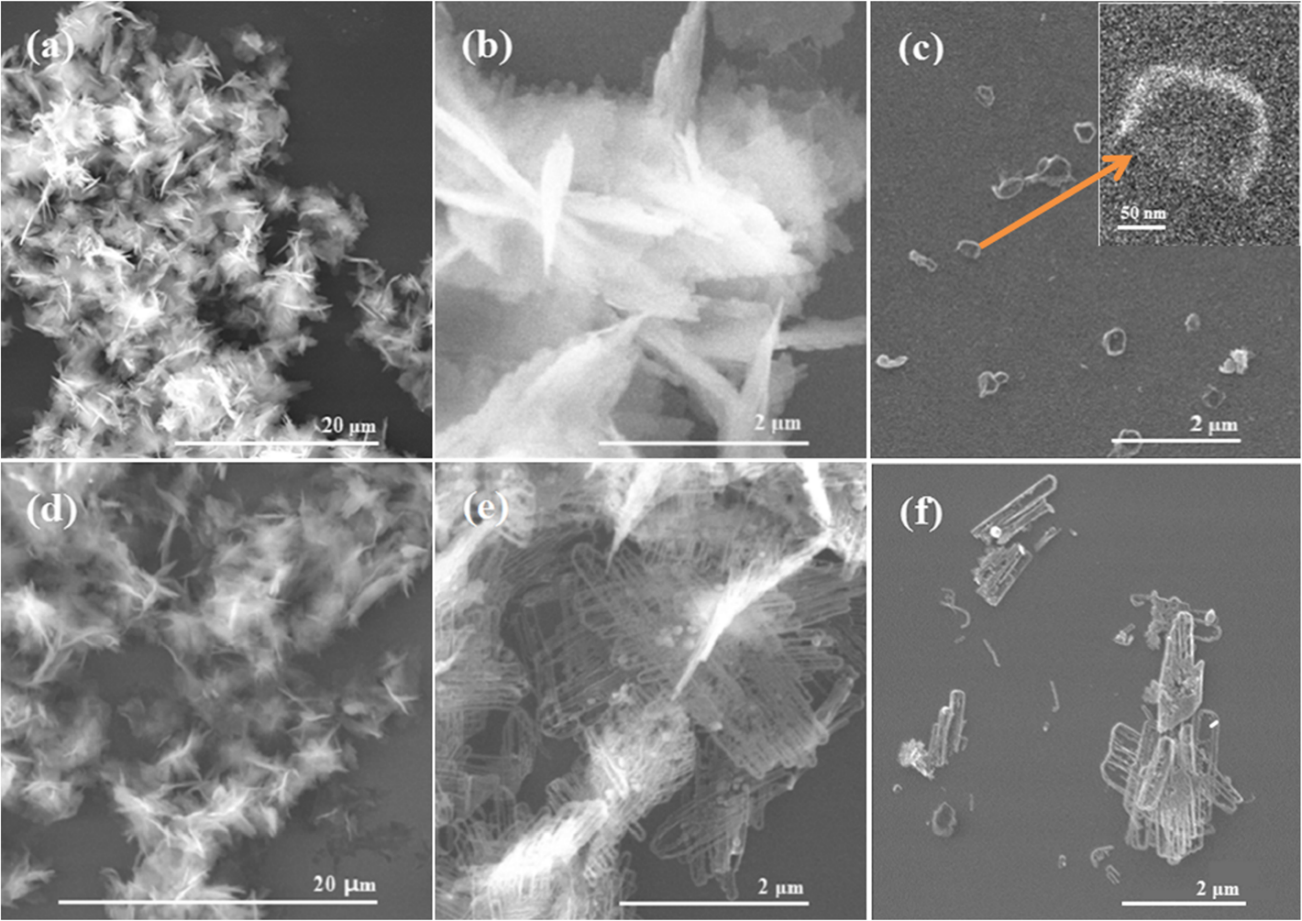
SEM images of ZnO samples from 0.01 M Zn(OAc)2·nH_2_O solution at 170 °C with reaction times of (**a**–**c**) 12 min and (**d**–**f**) 2.5 h. The inset of (**c**) is the local magnification of a nano-ring morphology

In order to elucidate possible growth mechanism of ZnO nano-clip formation, we also performed SEM observations on as-obtained early ZnO precipitation at reaction time of 12 min from 0.01 M solution at 170 °C. Figure 4 shows SEM images of ZnO samples with various reaction times of 12 min and 2.5 h.

Under low magnification view (× 5000), ZnO samples obtained at 12 min and 2.5 h exhibit similar morphologies with feather-like aggregates in Fig. 4a, d. Further increasing the magnification (× 50,000), for a 12-min sample, we cannot observe clear features and details in Fig. 4b; however, for a 2.5-h sample, accumulated nano-clips can be seen clearly in Fig. 4e. It is worth noticing that the early morphology of nano-clip such as nano-ring or half-ring has been found in the 12-min sample in Fig. 4c. This is an important hint to explain the formation mechanism of ZnO nano-clips. Moreover, we also recognize some parts of nano-clips in the 2.5-h sample, such as nanowire, nano-stick, and unclosed clip in Fig. 4f.

**Fig. 5:**
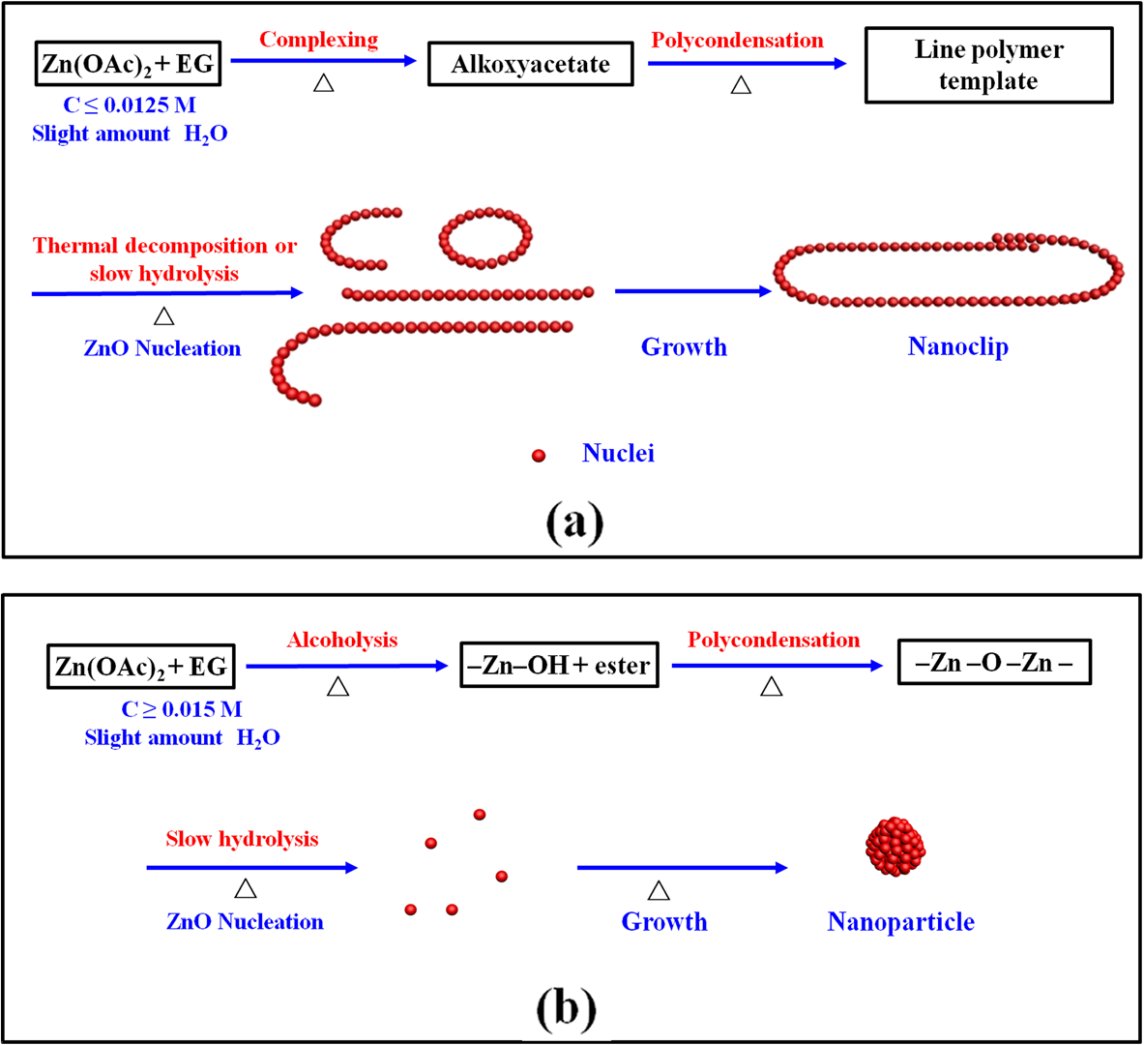
Evolution schematics of (**a)** ZnO nano-clip and (**b**) ZnO particle formation by two possible polyol-mediated routes

In our ZnO nano-clip preparation process, the Zn(OAc)_2_·nH_2_O solution concentration is 0.01 M and evidently lower than most references [23, 24, 28–30]; meanwhile, no extra water or alkaline such as NaOH or capping agent of polyvinyl pyrrolidone (PVP) are added into 5 mL EG solvent. Moreover, our used Zn source contains relatively less water of hydrate (*n* < 2) due to water loss caused by longer storage. The possible formation of ZnO nano-clips can be described as follows:

First, Zn(OAc)_2_·nH_2_O dissolves in EG solvent in around 1 min at 170 °C. Zinc acetate hydrate reacts with EG and forms the intermediate precursor of alkoxyacetate complex such as Zn(OAc)(OCH_2_CH_2_OH)_x_ by partly replacing acetate anions and water molecules (Eq. 1), as confirmed by FTIR spectra in Fig. 2c. The formation of the coordination bonds between the Zn^2+^ and the solvent of diethylene glycol (DEG) and EG has also been observed in several previous works [24, 28, 29]. Poul et al. have detected the alkoxyacetate complex existence of Zn(OAc)_3_(OCH_2_CH_2_OH) and Zn_3_(OAc)_4_(O(CH_2_)_2_O(CH_2_)_2_O) [34, 35]. Subsequently, alkoxyacetate complexes continue to polymerize and form a line polymer (Eq. 2). Acetate and EG acts as a bridging ligand allowing polymerization to occur. The FTIR spectra of as-prepared ZnO nano-clips also manifest the bridging bonding mode in Fig. 2c. Here, the line polymer just like a template induces the growth of ZnO nanocrystals along the long chain through thermal decomposition or slow hydrolysis so as to get a ZnO nanowire and nano-ring. After enough reaction time (≥ 1 h), the ZnO nano-clips from the ZnO nanowire and nano-ring are formed at last as shown in Fig. 5a.

The effect of other processing parameters such as reaction temperature, additives, solvent such as PVP, and Zn sources on the formation of ZnO nano-clips has been illustrated in Additional file 1. The nonhydrolytic alcoholysis reaction between Zn(OAc)_2_·nH_2_O and EG begins to predominate in ZnO nanocrystal fabrication [36, 37]. The H_2_O amount and OH^−^ concentration have important influence on the morphology and grain size of polyol-mediated ZnO products [23, 24, 27–30]. The high hydrolysis ratio (> 50) in EG leads to the hydroxyacetate formation [23]. Based on the literature reports [23, 24, 26], hydroxyacetate favors the formation of ZnO nanoparticles under these conditions. The –Zn–OH is formed by an alcoholysis route based on ester-elimination reaction (Eq. 3), then the polycondensation of –Zn–OH and –Zn–O–Ac or –Zn–OH leads to the progressive development of the ZnO nuclei by splitting off acetic acid or H_2_O (Eqs. 4 and 5), which might be concomitant with the slow hydrolysis reaction [28]. Equation 5 is equal to the forced hydrocondensation proposed by Gaudon et al. [27]. Finally, the ZnO nuclei grow larger to form ZnO nanocrystals. These nanocrystals aggregate to spherical or elliptical nanoparticles as shown in Fig. 5b. It is competitive between the two kinds of polyol reaction routes along with the change of processing parameters.
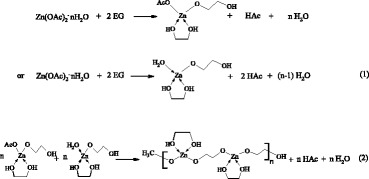

3 $$ \hbox{--} \mathrm{Zn}\hbox{--} {\mathrm{OOCCH}}_3+\mathrm{H}\hbox{--} {\mathrm{OC}}_2{\mathrm{H}}_4\mathrm{OH}\to \hbox{--} \mathrm{Zn}\hbox{--} \mathrm{OH}+\mathrm{H}\mathrm{O}\hbox{--} {\mathrm{C}}_2{\mathrm{H}}_4\hbox{--} {\mathrm{OOCCH}}_3 $$

4 $$ \hbox{--} \mathrm{Zn}\hbox{--} \mathrm{O}\hbox{--} \mathrm{H}+{\mathrm{CH}}_3\mathrm{COO}\hbox{--} \mathrm{Zn}\hbox{--} \to \hbox{--} \mathrm{Zn}\hbox{--} \mathrm{O}\hbox{--} \mathrm{Zn}\hbox{--} +{\mathrm{CH}}_3\mathrm{COO}\mathrm{H} $$

5 $$ \hbox{--} \mathrm{Zn}\hbox{--} \mathrm{O}\hbox{--} \mathrm{H}+\mathrm{H}\hbox{--} \mathrm{O}\hbox{--} \mathrm{Zn}\hbox{--} \to \hbox{--} \mathrm{Zn}\hbox{--} \mathrm{O}\hbox{--} \mathrm{Zn}\hbox{--} +{\mathrm{H}}_2\mathrm{O} $$

## Conclusions

Intriguing ZnO nano-clips with better monodispersion were prepared by a simple polyol-mediated route for the first time. The effect of solution concentration on the formation of ZnO nano-clips has been investigated deeply. We prove that the Zn(OAc)_2_·nH_2_O can react with EG without added water or alkaline, producing pure ZnO phase with polycrystalline wurtzite structure at 170 °C. The shape of ZnO nano-clips keeps constant with improved crystalline quality after annealing at 400–600 °C. The possible growth mechanism based on a competition between complexing and alcoholysis between Zn(OAc)_2_·nH_2_O and EG has been proposed. When the solution concentration is ≤ 0.0125 M in 5 mL solution at 170 °C, the complexing and polymerization reactions predominate, mainly elaborating ZnO nano-clips. When the solution concentration is ≥ 0.015 M, the alcoholysis and polycondensation reactions become dominant, leading to ZnO particle formation with spherical or elliptical shapes. Due to special nanostructures and larger specific surface area, ZnO nano-clips are a promising material as photocatalyst for degrading the harmful pollutants in waste water and gas, anode material of lithium battery or supercapacitor for electrochemical energy storage, and sensor for detecting dangerous gas.

## Additional file

Additional file 1: Effect of processing parameters on ZnO morphology. (DOCX 740 kb)

## Abbreviations

BET: Brunauer-Emmett-Teller
DEG: Diethylene glycol
EG: Ethylene glycol
FTIR: Fourier-transform infrared spectra
FWHM: Full width at half maximum
HRTEM: High-resolution transmission electron microscopy
NIR: Near infrared ray
PVP: Polyvinyl pyrrolidone
SEM: Scanning electron microscopy
TEM: Transmission electron microscopy
TG-DTG: Thermogravimetry-differential thermal gravity
UV: Ultroviolet
XRD: X-ray diffraction
