# Supplementary material for: Fabrication and Characterization of ZnO Nano-Clips by the Polyol-Mediated Process
Source: Nanoscale Res Lett. 2018 Feb 9;13:47. doi: 10.1186/s11671-018-2458-9 (PMC5807257; doi:10.1186/s11671-018-2458-9)
Supplement: Supplementary file 1 — Effect of processing parameters on ZnO morphology. (DOCX 740 kb) [file 11671_2018_2458_MOESM1_ESM.docx]

**Supplementary Materials**

**Fabrication and Characterization of ZnO Nano-clips by the Polyol-mediated Process**

Mei Wang, Ai-Dong Li*, Ji-Zhou Kong, You-Pin Gong, Chao Zhao, Yue-Feng Tang, and Di-Wu

National Laboratory of Solid State Microstructures, Department of Materials Science and Engineering, College of Engineering and Applied Sciences, Collaborative Innovation Center of Advanced Microstructures, Nanjing University, Nanjing 210093, People’s Republic of China

E-mail: [adli@nju.edu.cn](mailto:adli@nju.edu.cn)

**Effect of processing parameters on ZnO morphology**

1.1 The reaction scale-up and reaction temperature effect

In addition, we also explored the reaction scale-up effect. At 0.01 M and 170 ^o^C for 2 h, when increasing the reaction solvent amount from 5 mL to 40 mL, the reaction rate becomes slow due to more reactant of Zn(OAc)_2_·nH_2_O. The dissolving time of reactant in EG extends from 1 min to 5 min, and the tumid time from 6-7 min to 10-12 min. Finally, the ZnO products become uniform spheres of ~200-235 nm (Figure S1(a)). The BET specific surface area of as-prepared sphere samples is 23.6 m^2^/g. After 400 ^o^C anneal, the specific surface area reduces to 16.8 m^2^/g due to the enhanced sphere density and size.


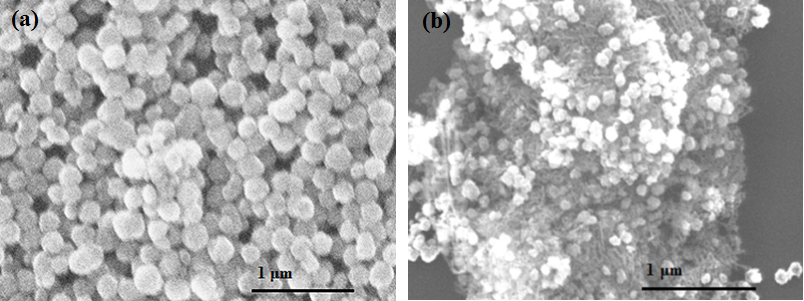


Figure S1 SEM images of ZnO samples under (a) 0.01 M, 40 mL, 170 ^o^C, and (b) 0.01 M, 5 mL, 180 ^o^C.

For 0.01 M solution, we also studied the reaction temperature effect from 170 ^o^C to 140 ^o^C, 160 ^o^C, and 180 ^o^C for 1hr. The ZnO nano-clip samples can be fabricated at 140-180 ^o^C. The reaction rate is related to temperature. At 140 ^o^C, the solution tumid time is 15 min. When temperature rises to 160 ^o^C and 180 ^o^C, the tumid time becomes 7.5 min and 4 min, respectively. Figure S1(b) shows the ZnO products of 180 ^o^C, there are more sphere ZnO particles compared to the products of 170 ^o^C. It illustrates that the products at higher temperature contain relatively more nanoparticles than the ones at lower temperature.

1.2 Additive effect

For 5 mL, 0.01 M Zn(OAc)_2_·nH_2_O solution, the impact of slight additives of 8.4 mg PVP, 4μL hydrochloric acid, 3.5 mg NaCl,1.5 mg MnCl_2_·6H_2_O, and a drop of NH_3_·H_2_O on morphology has been examined at reaction temperature of 170 ^o^C for 2 h. Figure S2 shows SEM images of ZnO samples with various additives. Except NH_3_ · H_2_O, ZnO clip-like shapes basically remain unchanged after adding slight PVP, HCl, NaCl, and MnCl_2_·6H_2_O, although some morphology difference can be recognized in Figure S2(a)-(d), such as unobvious gap or width of clips. In previous studies related to Refs. [1-2], PVP capping agent is beneficial to either form nano-rod structure, or decrease the nanoparticle size thus increase the surface area. But, in our study, with adding PVP, the monodispersity and shape integrity of ZnO nanoclip both become worse than that without adding PVP, as shown in Figure S2(a).


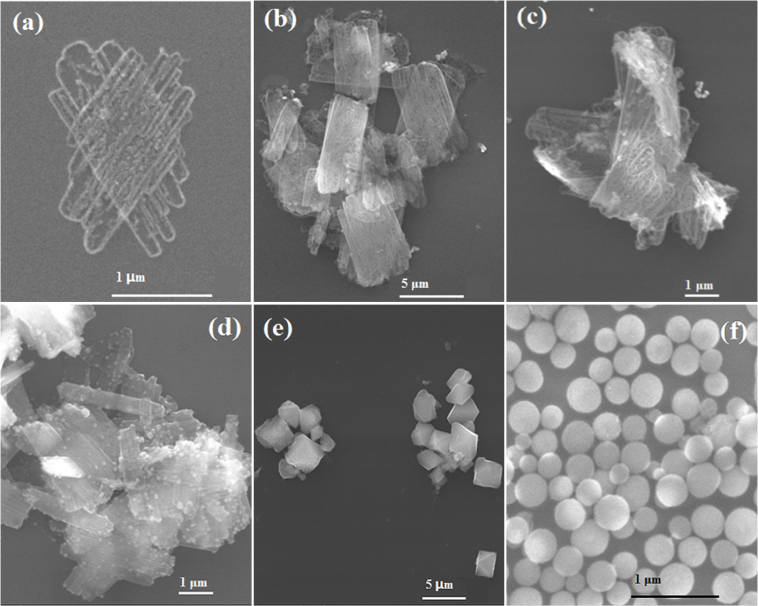


Figure S2 SEM images of ZnO samples with additives of (a) PVP, (b) hydrochloric acid, (c) NaCl, (d) MnCl_2_·6H_2_O, and (e) NH_3_·H_2_O. (f) SEM image of ZnO samples derived from DEG solvent.

In addition, after adding NaCl, the reaction becomes slow and the solution turns tumid after 35 min. Usually, the milky white precipitation is formed at around 7 min. While adding a drop of NH_3_·H_2_O into Zn(OAc)_2_·nH_2_O solution, some intriguing ZnO tetragonal cones with size of 2-3 μm can be obtained in Figure s2(e). Dakhlaoui et al. reported that the increase of the alkaline ratio results in a significant change of the ZnO morphology from conical, nanorod-like and elliptical shapes to spherical ones using DEG as solvent and NaOH as additive with high solution concentration of 0.5 M [3]. At lower alkaline ratio of 0-1.5, conical shapes with several tens to several hundred nanometer sizes can be observed, because the lower alkaline ratio is beneficial to the stretched growth along the c-axis of the ZnO. Herein, the additive of NH_3_·H_2_O is weak basic and the alkaline ratio in our solution is very small (~6.5×10^-5^), however, NH_3_·H_2_O contains more water, leading to the higher hydrolysis ratio (the molar ratio of water to metal) of ~20 in EG. Finally, the ZnO samples show tetrahedral conical shapes instead of nanoclips.

1.3 Solvent and zinc source effect

The solvent and zinc source effect has been probed. The EG solvent was replaced with DEG and TEG to obtain 5 mL, 0.01 M Zn(OAc)_2_·nH_2_O solution. The reaction temperature kept at 170 ^o^C for 2 h. The ZnO products derived from DEG are very smooth spheres with diameter of ~250-385 nm, as seen in Figure s2(f). The ZnO products from TEG are smaller spheres with diameter of ~150 nm. Simultaneously, the reaction becomes slow. The solution turns tumid at about 30-35 min for DEG and TEG solvents rather than 6-7 min for EG one. Compared to EG (b.p. of 197 ^o^C), DEG and TEG have higher boiling points of 245 ^o^C and 285 ^o^C, respectively. So the reaction temperature of 170 ^o^C is a little lower in comparison with their boiling points, resulting in slower reaction rate. The polyol solvent-dependent ZnO morphology and size have also been observed using Zn(OAc)_2_·2H_2_O in Refs. [4,5]. For example, the shape of the ZnO nanoparticles changed from spherical (~19 nm, EG), spherical and rod (~ 39 nm, DEG) to ‘diamond’ like structure (~69 nm, tetraethylene glycol (TTEG)) in 1 M Zn(Ac)_2_·2H_2_O solution at 160 ^o^C [5].

When the Zn acetate was substituted by Zn(NO_3_)_2_·6H_2_O and ZnSO_4_·7H_2_O, other processing conditions such as solution concentration, EG solvent volume, and reaction temperature/time were fixed to 0.01 M, 5 mL, and 170 ^o^C/2 h, respectively. There is no ZnO precipitation formation using Zn(NO_3_)_2_·6H_2_O and ZnSO_4_·7H_2_O as Zn source, i.e. no chemical reaction between Zn nitrate/Zn sulfate and EG. This result of ZnSO_4_·7H_2_O source is consistent with the literature using EG or DEG [4,6]. However, in Ref. 5, the Zn(NO_3_)_2_·6H_2_O give rise to brown ZnO in DEG due to some degree of reduction of nitrate anions, quite different from our result. It is possibly related to the solvent, reaction temperature, and solution concentration.

References

1. Zhou Q, Chen W, Xu L, Peng S (2013) **Hydrothermal synthesis of various hierarchical ZnO nanostructures and their methane sensing properties**. *Sensors* 13: 6171-6182

2**.** Javed R, Usman M, Tabassum S, Zia M (2016) **Effect of capping agents: structural, optical and biological properties of ZnO nanoparticles.***Applied Surface Science* 386: 319-326

3. Dakhlaoui A, Jendoubi M, Smiri L S, Kanaev A, Jouini N (2009) **Synthesis, characterization and optical properties of ZnO nanoparticles with controlled size and morphology**. *Journal of Crystal Growth* 311: 3989-3996

4. Jézéquel D, Guenot J, Jouini N, Fiévet F (1995) **Submicrometer zinc oxide particles: Elaboration in polyol medium and morphological characteristics.** *J. Mater. Res.* 10: 77-83

5. Chieng B W, Loo Y Y (2012) **Synthesis of ZnO nanoparticles by modified polyol method**. *Materials Letters* 73: 78-82

6. Poul L, Ammar S, Jouini N, Fiévet F (2003) **Synthesis of Inorganic Compounds (Metal, Oxide and Hydroxide) in Polyol Medium: A Versatile Route Related to the Sol-Gel Process.** *Journal of Sol-Gel Science and Technology* 26: 261-265
